# Silk-fibronectin protein alloy fibres support cell adhesion and viability as a high strength, matrix fibre analogue

**DOI:** 10.1038/srep45653

**Published:** 2017-04-05

**Authors:** Matthew M. Jacobsen, David Li, Nae Gyune Rim, Daniel Backman, Michael L. Smith, Joyce Y. Wong

**Affiliations:** 1 Department of Biomedical Engineering, Boston University, 44 Cummington Mall, Boston, 02215, Massachusetts, USA

## Abstract

Silk is a natural polymer with broad utility in biomedical applications because it exhibits general biocompatibility and high tensile material properties. While mechanical integrity is important for most biomaterial applications, proper function and integration also requires biomaterial incorporation into complex surrounding tissues for many physiologically relevant processes such as wound healing. In this study, we spin silk fibroin into a protein alloy fibre with whole fibronectin using wet spinning approaches in order to synergize their respective strength and cell interaction capabilities. Results demonstrate that silk fibroin alone is a poor adhesive surface for fibroblasts, endothelial cells, and vascular smooth muscle cells in the absence of serum. However, significantly improved cell attachment is observed to silk-fibronectin alloy fibres without serum present while not compromising the fibres’ mechanical integrity. Additionally, cell viability is improved up to six fold on alloy fibres when serum is present while migration and spreading generally increase as well. These findings demonstrate the utility of composite protein alloys as inexpensive and effective means to create durable, biologically active biomaterials.

In order to design improved biomaterials for tissue engineering purposes, inspiration can be found in the native tissue environment as a model system. Simply put, tissues are comprised of complex networks of cells suspended in extracellular matrix (ECM) elements. Large components of this matrix consist of fibrous protein structures including fibronectin (Fn) and collagen, which are ubiquitous throughout the body. Cells associate with these protein fibres via specific molecular recognition sites that mechanically link intracellular and extracellular environments. This in turn allows cells to exert forces on their environment – necessary for viability in most adult cell lines. However, recapitulating this type of fibrous protein network in order to fabricate an *in vitro* ECM is a difficult prospect due to the difficulty in synthetically producing Fn^1^,^2^ and collagen^3^,^4^ in their native fibre forms. Even though ECM-derived scaffolds have been sourced natively^5^, they are often processed through crushing, cross-linking, freeze drying, and/or gelation such that they no longer appropriately emulate the truly dynamic mechanical environment of a fibrous network. While tissue decellularisation techniques have improved the conservation of this dynamic environment^6^, these scaffolds require availability of a tissue or organ donor source, which limits their throughput potential.

In this regard, silk fibres are excellent candidates for use as supporting scaffolding materials because they are naturally spun, biological fibres that have favourable mechanical properties including high tensile strength, stiffness, toughness, ductility^7^, and they exhibit good biocompatibility^8^. When dissolved, silk from the silkworm *Bombyx mori* can be reprocessed as regenerated silk fibroin (SF) into many forms including films^9^, gels^10^, and fibres, both of the nano^11^- and micro-scales^12^. However, silk is a poor material with regard to biological interactivity^13^ in that it has a very limited capability to bind and sustain cells in contact with it. Therefore, it requires supplemental modification in order to improve this capability such that it can effectively engage with cells in its local environment and successfully integrate itself as a biomaterial in tissues.

To this end, efforts have been directed to treat or condition SF in such a way as to improve its biocompatibility or material properties. For improvements in biocompatibility, silk has been mixed into various forms with additive compounds including collagen to generally improve cell interactions^14^,^15^,^16^, hydroxyapatite to promote osteogenic activity^17^,^18^, and poly(ethylene glycol) to prevent surface fouling^19^. Its material properties have likewise been manipulated through additions such as carbon nanotubes for hardening^20^ and poly(lactic-co-glycolic acid) to modify biological degradability^21^. Efforts have also been made to create silk-based chimeric proteins with sequences sourced from elastin^22^, collagen^23^,^24^,^25^, and Fn, particularly the RGD (Arg-Gly-Asp) cell binding domain^26^,^27^,^28^, all with the intent to improve native silk bioactivity for biological applications. However, recombinant protein production comes with a substantial methodological barrier while only recapitulating a small degree of function from their parent proteins, such as the adhesive and viability differences of the RGD peptide versus full-length Fn^29^,^30^. Additionally, these experimental modifications, where successful, have focused almost exclusively on hydrogel, film, and electrospun nano-fibre silk constructs, which all exhibit considerable mechanical limitations compared to wet-spun, micro-scale fibres^31^.

This is due, in part, to the wet-spun fibre spinning methodology being a much closer analogue to the native spinning process in spiders and silkworms in form and scale by recapitulated key aspects of the process such as diameter tapering, shearing, and ion exchange^12^,^32^,^33^. Yet, despite the potential material advantage afforded by these types of fibres, there have not been many efforts to modify such constructs into more biologically favourable biomaterials. Yang *et al*.^34^ presented a hybrid mixture of SF with a mussel adhesive protein modified with RGD, yet the wet-spun products were only characterized by their mechanical properties whereas their cell characterization studies focused on their electrospun products. Zhang *et al*.^35^ produced a hybrid SF wet-spun fibre with a polysaccharide plant extract from *Bletilla striata*, but similarly did not extend their analysis to mechanically characterising nor quantifying the degree of bioactivity or improved cellular interactions. As such, there is a need to demonstrate that modified wet-spun fibres can both retain greater mechanical properties, such as strength and stiffness, compared to the other reconstituted forms of SF biomaterials while also providing improved cellular interactions that SF alone lacks.

Therefore, in this work we present a hybrid silk fibroin-fibronectin (SF-Fn) protein alloy fibre fabricated via wet-spinning techniques that can meet mechanical and cell adhesive criteria that will enable its use as a surrogate structure for ECM fibres. Similar to their namesake of metal alloys, protein alloys are chemically^36^ or physically^37^ bonded mixtures of component proteins that synergize elements of their constitutive parts when processed into a usable form. Through wet spinning, we gain flexibility to incorporate new elements without sacrificing the biomimetic nature of the approach, which allows us to incorporate Fn molecules within the silk superstructure rather than simply being a surface modification. The intrinsic incorporation of the entire Fn protein provides additional interaction potential with cells and the native matrix environment of tissues beyond simple adhesion afforded by the RGD domain. This is due to the ability of Fn to bind many cell adhesion and signalling molecules including more than 20 growth factors^30^. Furthermore, Fn possesses complex integrin-binding properties that are not captured by RGD alone^29^. Consequently, a fibre alloy that exhibits both the mechanical advantages of SF and the cell interactivity of the native ECM offers great potential for use in tissue scaffolding applications for eventual use *in vivo*.

## Results

### Fibronectin incorporation into the silk fibre structure

**Figure 1:**
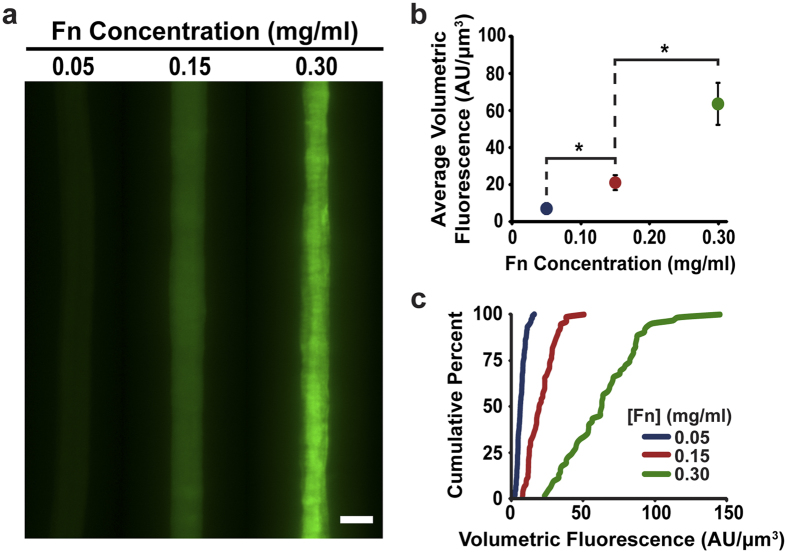
Incorporation of fluorescently labelled Fn into the SF fibre. (**a**) At increasing doping concentrations, greater amounts of Fn are incorporated into the alloy fibres. (**b**) The total average loading follows an approximately linear behaviour as doping concentration increases (N = 5). (**c**) There is a respective decrease in the homogeneity of the Fn distribution along the length of the fibres as doping increases. Scale bar is 15 μm. *Indicates statistical significance.

In order to verify the integration of Fn into the SF fibre superstructure, fluorescent images along the length of fibres doped with fluorescently labelled Fn were taken. Epifluorescence data demonstrate that Fn is successfully incorporated throughout the entire volume of the SF fibre (Fig. 1a), which is confirmed by confocal imaging (Fig. S1). The quantity of fibre-bound Fn varies with the doping concentration and shows a measured response. Higher doping concentrations yield greater mean intensities that are statistically significant (*P* < 0.0001 between 0.05 and 0.15 mg/ml Fn concentrations in the fibre; *P* < 0.0001 between 0.15 and 0.30 mg/ml), following an approximately linear relationship (Fig. 1b). However, higher quantities of incorporated Fn also lead to decreasing homogeneity of its distribution along the length of the fibres. Figure 1c shows the cumulative distribution plot of the fibres, which describes the probability that a pixel within the fibre will have a value less than or equal to its respective value on the x-axis. The curves for the lower concentrations of 0.05 and 0.15 mg/ml have greater slopes (*m* = 0.13 and *m* = 0.038, respectively), indicating more uniformity of the distribution of volumetric fluorescence throughout the fibre compared to the lower slope of the 0.30 mg/ml concentration (*m* = 0.013) signalling a wider distribution of fluorescence intensities.

### Mechanical properties of the spun fibres

**Figure 2:**
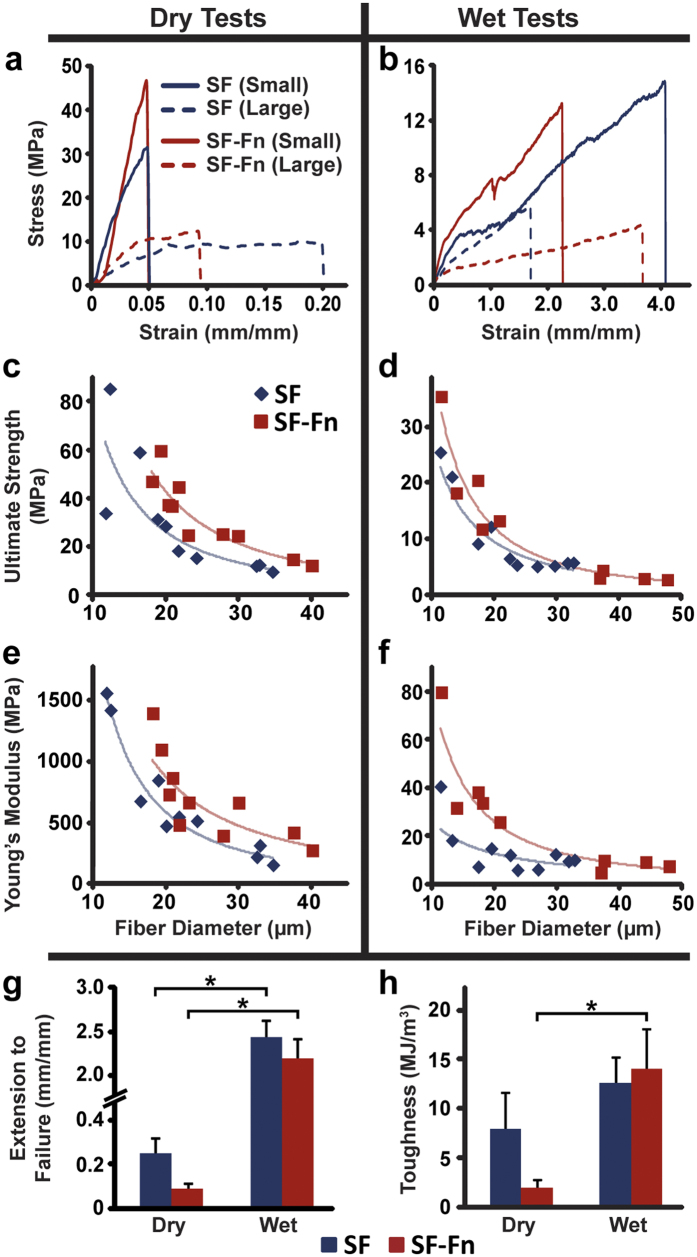
Mechanical data of the control and alloy fibres in dry and wet conditions. (**a**,**b**) Representative stress-strain curves for small diameter (less than 20 μm) and large diameter (greater than 30 μm) fibres are shown from which the mechanical properties are calculated (N = 10 for all except wet SF-Fn, N = 9). (**c–f**) Raw data of ultimate strength and Young’s modulus plotted against diameter demonstrate a dependent relationship. Power regression lines fitted to the data show a general right-shifting of the curve for the alloy fibres. (**g**) Extension to failure is not coupled to fibre diameters and is generally lower for alloy fibres. (**h**) Material toughness does not change significantly from control to alloy fibres. *Indicates statistical significance.

To determine if incorporation of a sufficient quantity of Fn into the SF structure to elicit greatly increased cell adhesion would disrupt the fibre’s mechanical integrity, we performed tensile tests on fibres under dry and wet conditions (Fig. 2a,b). Self-comparison of the dry and wet conditions for the control and alloy fibres, respectively, shows significant differences in ultimate strength (Fig. 2c,d) (*P *=* *0.027, *P *=* *0.0037), Young’s modulus (Fig. 2e,f) (*P *=* *0.0031, *P *=* *0.011), and extension to failure (Fig. 2g) (P < 0.0001, *P *<* *0.0001). However, toughness only exhibits a statistically significant difference in SF-Fn alloy fibres (Fig. 2h) (P = 0.021, compared to *P *=* *0.33 for SF fibres). For both fibre types, extension to failure increases by approximately 10-fold or more on average in wet tests while simultaneously experiencing about a two-thirds reduction in ultimate strength.

When comparing the SF and SF-Fn fibres directly, ultimate strength and Young’s modulus (Fig. 2c–f) exhibit a diameter dependence that follows a power regression. The exponents of the regressions in Fig. 2b, representing the slopes of the fitted log-linear lines, for ultimate strength of SF fibres are −1.650 and −1.546; for SF-Fn fibres, −1.720 and −1.803 for dry and wet tests, respectively. Exponents of Young’s modulus regression lines for SF fibres are −1.848 and −1.063; for SF-Fn fibres, −1.511 and −1.660 for dry and wet tests, respectively. The metrics demonstrate a right-shifting of the regression curve for the alloy fibres, indicating a strengthening and stiffening effect with Fn incorporation, which persists at high Fn concentrations as well (Fig. S2). Extension to failure (Fig. 2g) does not exhibit a correlation with fibre diameter, and while alloy fibres consistently exhibit lower extension to failure, there is no significant difference between the two fibre types in either dry (*P* = 0.058) or wet (*P* = 0.41) cases, though it is a slim margin between dry SF and SF-Fn. Because extension is decoupled from fibre diameter, material toughness is also independent of fibre diameters (Fig. 2h). Despite the alloy exhibiting a strengthening and stiffening behaviour compared to the control fibre in both dry and wet cases, the marginal losses of extension reduce the total overall energy absorbed by alloys relative to the control while dry, though not by a significant amount (*P* = 0.16). However, when wetted, the alloy fibres demonstrate equal material toughness to the control SF fibres (*P* = 0.78).

### Cellular attachment to fibres without serum

**Figure 3:**
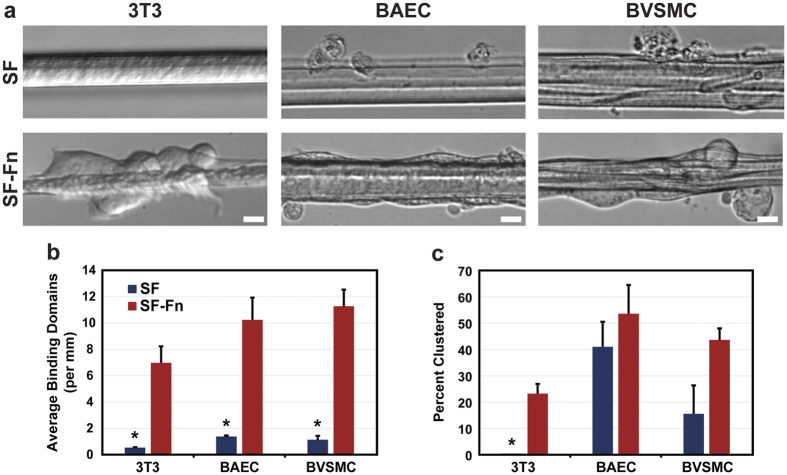
Cell attachment on control and alloy fibres in serum-deprived media. (**a**) Images shown for three cell types seeded onto the two fibre systems after washing away unattached cells and debris after 24 hours of incubation (N = 3). (**b**) For all cell types, cell adherence to the fibres is significantly improved by the incorporation of Fn into SF. Additionally, cell appearance on SF-Fn alloys generally lack notable signs of cell death, as defined earlier such as compromised membranes, that are much more common with cells associated with the SF fibres at this seeding density. (**c**) Cell-cell interactions on the fibres are also improved through Fn incorporation leading to higher clustering at attachment loci, though not significantly higher for BAECs and BVSMCs. Scale bars are 15 μm. *Indicates statistical significance.

As a means to assess the cell adhesive properties gained from incorporating Fn into the SF fibre framework, cells were cultured on alloy and control fibres for 24 hours in media without serum. Imaging analysis reveals that the intrinsic capability of SF to bind cells is indeed increased by incorporating Fn (Fig. 3a). Cells cultured on SF fibres yield approximately one binding locus per millimetre, which is defined as each location along the fibre where a unique cell is directly bound. In contrast, in the case of alloy SF-Fn fibres, cells attach readily, even in the absence of serum (Fig. 3b), with binding increasing greater than 6-fold for 3T3s (*P *=* *0.0070) and 10-fold for BAECs (*P* = 0.0065) and BVSMCs (*P* = 0.0015). Cell-cell interactivity at the attachment loci also trend in the positive direction for all cell types, shown in Fig. 3c as the percent of all loci that have more than one cell attached at the same location. However, only the 3T3 clustering (*P *=* *0.0033) demonstrates a statistically significant change in this metric, which is likely a consequence of the fact that BAECs (*P* = 0.44) and BVSMCs (*P* = 0.074) demonstrate a high degree of clustering compared to 3T3s even in the control SF seeding case.

### Cell migration and viability on fibres

**Figure 4:**
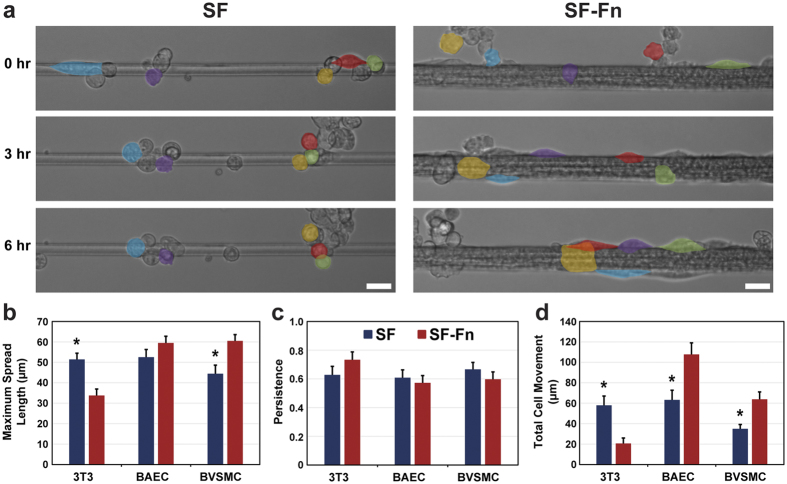
Migration behaviour of cells on control and alloy fibres. (**a**) Representative images of points during the 12 hour time-lapse showing a general characteristic shared by the cell types. False coloration is added to show the migration paths of specific cells. On SF fibres, cells tend to migrate into clusters, which often would lead to detachment from the fibre into the cell cluster. Contrary to this, on SF-Fn fibres, cells from cell clusters tend to detach from the cluster to attach to the fibres and still maintain connectivity with neighbouring cells. (**b**) Cell spread length increases on SF-Fn fibres significantly for BVSMCs and not significantly for BAECs. 3T3s show the opposite trend in a significant manner. (**c**) None of the cells tended toward a specific direction nor did they maintain their velocity. This was unaffected from control to alloy fibres. (**d**) BAECs and BVSMCs both show significantly higher total cell motion on SF-Fn fibres while 3T3s again show the opposite trend, also significantly. Scale bars represent 25 μm, N = 30 for all cell and fibre types. *Indicates statistical significance.

To determine if incorporation of Fn into SF fibres increases the longevity of cellular interactions and survival on fibres, we took time-lapse images over 12-hours of interactions with images taken every 10 minutes. Prior to time-lapse microscopy, cells were seeded onto fibres and incubated for 1 hour in serum-enriched media. Figure 4a shows representative images of the general behaviour of cells during the time-lapse. Cells on SF fibres would preferentially cluster together rather than staying attached to the fibre, leading to many terminal detachments or arrested migration of attached cells. On SF-Fn fibres, the trend is reversed where free clusters of cells that attach to the fibre tend to disassociate from the cell cluster in favour of binding to the fibre and continued migration. The terminal detachment rate on SF-Fn is consequently much lower than that of the SF fibres for all cell types. Similarly, all cells show a lack of directional preference during migration (*P *=* *0.19, *P* = 0.63, and *P* = 0.33 for 3T3s, BAECs, and BVSMCs, respectively), often changing directions throughout the period (Fig. 4c). However, for the metrics of maximum cell spread length (Fig. 4b) and total cell movement (Fig. 4d), 3T3s show an opposing trend to that of the BAECs and BVSMCs. The maximum extension length of cells on SF-Fn fibres is increased for both BAECs (*P* = 0.17) and BVSMCs (*P* = 0.031), although only significantly for BVSMCs, and both BAECs (*P* = 0.004) and BVSMCs (*P* = 0.001) travel significantly farther overall. 3T3s demonstrate a less active phenotype, spreading significantly less (*P* = 0.0002) and traveling significantly less distances (*P* = 0.0008). Despite this lower activity from 3T3s, the other cell types demonstrate that SF-Fn fibres generally lead to increased cell migratory activity.

**Figure 5:**
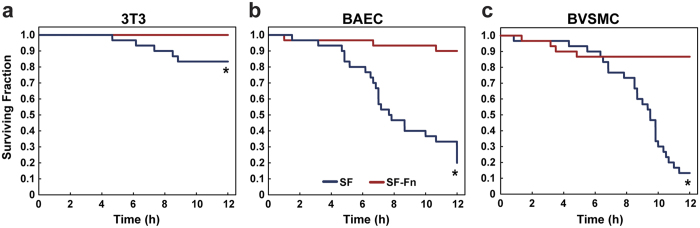
Survival profile of cells seeded in serum onto control and alloy fibres. Cell survival was tracked over the course of 12 hours in addition to migration and spreading behaviour as detailed in the methods. (**a**) 3T3 cells proved to be the most robust, with the lowest cell loss on both fibre systems of the three cell types. (**b**) BAECs predominantly detached terminally from the fibres rather than dying while attached. (**c**) BVSMCs showed the largest degree of cell death while remaining attached to the fibre. In all cases, SF-Fn fibres demonstrated a distinct survival advantage in both discouraging terminal detachment from the fibre as well as reducing the rate of cell death while attached to the fibre (N = 30). *Indicates statistical significance.

Cell survival was defined as outlined in the methods as cells that did not die or terminally detach from the fibre during the migration period (Fig. S5). As mentioned, cells on SF fibres exhibited a much greater propensity to detach or die over the 12-hour period. 3T3s proved the most resilient on SF fibres of the 3 cell types (Fig. 5a) only losing 17% of the tracked cells, whereas BVSMCs were the least viable losing 87% (Fig. 5c), and BAECs also showing low viability with 80% loss (Fig. 5b). In contrast, the highest fraction of death and detachment for SF-Fn fibres was observed from BVSMCs at 13.3%, then BAECs at 10%, and no death or detachment was observed for 3T3s. Log-rank tests of control vs alloy fibres show that SF-Fn fibres demonstrate significant improvement in cell viability for all cell types, including 3T3s (*P* = 0.018), although most markedly for BAECs (*P* < 0.0001) and BVSMCs (*P* < 0.0001).

## Discussion

Mimicking the ECM in both form and function is the most promising avenue of research inquiry to produce successful tissue scaffolds for biomedical purposes using *in vitro* techniques. In order to accomplish this, a reliable and high-throughput means of producing matrix fibre analogues will be paramount. In this work, we demonstrate a technique to create protein alloys of functional proteins using a simple spinning technique that produces fibres with hybrid functionality without necessitating sequence recombination at the genetic level. Specifically, we demonstrate that SF-Fn alloy fibres are capable of fulfilling the mechanical role of SF and the adhesive role of Fn in a single fibre and hold promise as potential scaffolding materials.

Our data suggest that incorporation of Fn into SF fibres is not only successful but also integrates functional characteristics of Fn and SF. It grants a significant survival advantage over SF fibres alone by encouraging cells to stay attached, spread, and motile. Furthermore, our cell-fibre interaction data suggest that SF fibres alone are very poor at both engaging and sustaining cells in culture even in the presence of serum and especially not in its absence. Therefore, we confirm previous studies demonstrating SF to have poor cell interaction capability without the aid of additives or enriched culture medium^13^,^38^. Modifying SF fibres into protein alloys through Fn addition gives the SF fibre the capability to interact with cells independent of its culture medium. In terms of tissue applications, these alloy fibres also provide mechanical stability, which together with cell adherence, activity, and viability constitute key requirements of a potential tissue scaffolding material.

The total quantity of Fn necessary to obtain this functional change is very low compared to the SF content of the fibre. On the low end, the mass ratio of SF:Fn is 1575:1, which represents an absolute concentration of 0.05 mg/ml of Fn that is a common concentration to treat surfaces for cell culture purposes. From this baseline value, the mass ratio was dropped as low as 75:1 to span two orders of magnitude and, yet, fibres are still able to be generated without issue. Accordingly, it is possible that cellular adhesion to the fibre could be improved further than reported here. As incorporated amounts of Fn increase, a proportional increase in the number of ligand binding sites on the fibre surface should occur as well, which can lead to additional interactive capacity of the alloy fibre with any cells in contact with it^39^ and more closely approximate a pure Fn fibre. This ability to quickly and simply incorporate functional amounts of entire exogenous proteins into SF fibres carries a distinct advantage over genetic hybridization and fusion protein creation via recombination, which can be prohibitively expensive and time-intensive processes. Such genetic modifications also lack any modularity as an entirely new gene must be integrated in order to change the functional output of the protein. On the contrary, this alloy system may be easily adapted to substituting components, such as collagen for Fn, demonstrating an even more significant throughput advantage.

Due to being a modified, wet-spun fibre, these alloy fibres maintain mechanical advantages over other similarly modified SF applications. Currently, the most common types of modified regenerated SF forms are hydrogels, films, and electrospun nano-scale fibres. The range of compressive stresses to failure and moduli of SF hydrogels varies between 0.01-3.0 MPa and 0.0005–6.5 MPa, respectively^10^,^15^. However, an important distinction to note for hydrogels is that their applications generally require them to bear load under compressive conditions rather than tensile loads and therefore serve a different purpose than fibres. A more direct comparison can be made with nano-scale fibre meshes that exhibit tensile moduli reported between 5-15 MPa with failure stresses between 0.5-4 MPa^20^,^40^. Our data show that alloy fibres have tensile moduli up to 1.5 GPa while dry and 80 MPa while wet, with failure strengths up to 60 MPa while dry and 35 MPa while wet. These values are mechanically superior to the other SF forms and are obtained using a simple spinning apparatus of either the shear extrusion method, which emulates the natural process of diameter tapering and protein shearing, or the microfluidic method, which emulates diameter tapering and ion exchange. For both systems, numerous conditions may be manipulated to improve the wet-spun fibres further, including concentrating the primary SF dope^10^, adding additional ions^41^, post-stretching the fibres^31^, and improving the shear stress profile during spinning^33^. Thus, our reported values can be read as the low end of the mechanical properties obtainable through wet-spinning processing. On the high end, several groups employing these processing manipulations have created silk fibres with strength values closer approximating those of native fibres, exceeding several hundred MPa^42^,^43^. Indeed, Yang *et al*.^34^ have reported strength values in the hundreds of MPa from their modified wet-spun silk fibres by virtue of using a highly concentrated silk dope and post-stretching the fibres.

Our data also demonstrate a diameter dependence on the mechanical properties of ultimate strength and elastic modulus. A common technique to improve SF fibre mechanics is post-spin drawing of the fibre to lengths up to five times the extruded length, which causes molecular realignment and leads to improved overall strength^31^,^44^. Our data support the conclusion that smaller diameter fibres have improved mechanical properties, but none of our materials have been subjected to post-spin drawing. Rather, extruded fibres vary in diameter between 10–50 μm as a result of varying the rate at which the focusing solution is flowed into the silk dope stream in the microfluidic device during spinning. This result supports the conclusion that the means to obtain higher strength, small diameter fibres, is not dependent on the physical stretching event accomplished by post-spin drawing. Instead, we support the hypotheses of Liivak *et al*.^32^ and Porter, Guan, and Vollrath^45^ that reduction in SF fibre diameter, regardless of cause, is what leads to improved mechanical behaviour. This is likely due to a higher degree of molecular alignment within the SF strand, but this hypothesis awaits future investigation, and we speculate that it may be apparent through measurements of the anisotropic properties of small versus large diameter wet-spun SF fibres.

Interestingly, the mechanical properties show a slight gain in material strength with respect to diameter with Fn added, which persisted at greater concentrations of Fn as well. This may infer that Fn is forming intermolecular β-sheets^46^ with the SF chains, which would mean that there is physical cross-linking occurring between the Fn and SF strands (Fig. S3). Indeed, β-strand exchange is believed to be an important contribution to the stabilization of Fn fibres^47^. It should be noted that Fn fibres are substantially weaker, with instantaneous slopes of stress versus strain curves reaching only a few MPa under hydrated conditions^48^. Additionally, the exceptionally long contour length of a single, folded Fn molecule, ~140 nm^49^, might allow entropic entanglement to bind it within the SF lattice structure. Therefore, in addition to ionic and hydrophobic/hydrophobic interactions, it is likely that several of these interactions comprise the integration assembly of the alloy with portions of Fn being physically cross-linked with the SF proteins and others being more loosely associated in the bulk or on the surface of the fibre.

For our purposes, Fn was selected as the functional protein to incorporate into the SF fibre because of its strong cell-binding capability and ubiquity in the body. However, as mentioned before, we submit that our findings are highly modular as there are many candidate proteins in the ECM that could be incorporated for other types of applications. Collagens, for example, comprise the most common proteins of the human body and are important structural proteins in the ECM. Type I collagen, like Fn, exists *in vivo* as a natural fibre; however, it is not easily recapitulated as such *in vitro*^50^ due to its highly ordered triple helical structure. Where collagen fibres have been recapitulated, post-spin cross-linking is often performed to prevent quick degradation of the material when exposed to collagenase activity *in vivo*, which can prove toxic to local environments^51^. As an alloy, the SF component could act as a surrogate structural element that would resist degradation through protease activity, thereby eliminating the need for chemical cross-linking. This could allow the collagen elements to provide their functional activity, just as the Fn in these alloy data, for a longer period of time. This would require eventual investigation into the degradation characteristics of the alloy fibres *in vivo*. This would give insight not only into how the fibres would degrade, but also whether or not function would be lost prior to that point or if the naturally low immunogenicity of SF^8^ would be altered through the process.

In addition to collagen, other matrix proteins such as laminin could be incorporated in order to create application-driven alloy materials. Because of the high degree of tunability in the mixing ratio of SF to a protein of interest, functional quantities of target proteins or several proteins can potentially be loaded into the fibres without disrupting fibre formation. In this regard, complex alloy fibres can be formed that would allow observation of the synergistic interactions caused by the different incorporated proteins. However, even with such modification, wet-spun fibres as presently constituted do not truly replicate the natural fibre environment of ECM. Generally, SF is a slow-degrading material *in vivo* that resists rapid degeneration, resorption, and modification by the cell environment surrounding it^52^. Therefore, localized cells may not be able to physically remodel the SF materials as they are able to do with natural ECM. This may alter their native *in vivo* behaviour, but can potentially be mitigated by driving down the diameters of SF alloy fibres toward the native range of ECM fibres, between 0.5–3 μm^5^. Future work should investigate whether the fibre spinning process can be tuned to accommodate this criterion while avoiding jeopardizing the mechanical advantages afforded by wet spinning.

## Conclusions

We have demonstrated that wet-spinning approaches enable the alloy assembly of SF-Fn fibres without detrimentally affecting the mechanical properties of the fibre. This type of protein alloy holds promise for fabricating biologically active and mechanically strong materials inspired by the ECM for tissue engineering applications, especially those with higher mechanical requirements than what current materials are capable of withstanding. The fabrication process has flexibility in that multiple functional proteins can potentially be incorporated into the alloy based on design needs in order to generate fibre characteristics that are directed toward tissue-specific demands. Therefore, these wet-spun alloy fibre constructs can serve as good templates to be organized into higher order structures for use in various scaffolding applications including neural, bone, and vascular tissue engineering.

## Methods

### Preparation of silk alloy solution

SF was sourced from degummed cocoon silk of *Bombyx mori* and prepared into an aqueous solution following an established protocol^53^, which was provided by the Kaplan laboratory of Tufts University. The weight percent concentration of the final solution was determined by weighing the residual solid before and after water evaporation. Whole Fn protein was isolated from human blood plasma (Valley Biomedical, Winchester, VA) and filtered using a two column chromatography separation^54^. Work done with human-sourced plasma was done according to approved protocol number 16–619 from the Institutional Biosafety Committee of Boston University in accordance with all relevant safety guidelines and regulations. Eluted Fn was stored in 1x phosphate-buffered solution (PBS) (Corning Inc., Corning, NY). A portion of the isolated Fn was labelled with Alexa Fluor 633 (Life Technologies, Grand Island, NY) via succinimidyl ester chemistry. Final solution concentrations were determined through UV spectroscopy. The protein alloy solution was then prepared by doping the Fn solution into the SF solution through pipetting and mild vortexing. The final protein concentrations of the Fn in solution were 0.05, 0.15, 0.30, and 0.75 mg/ml for the various experiments. The respective SF concentrations of the mixed proteins were 7.5, 6.6, and 5.3 wt% for samples with fluorescently labelled Fn and 7.9, 7.6, and 6.0 wt% for samples with unlabelled Fn. The 0.75 mg/ml Fn concentration was not used for fluorescence analysis; likewise, the 0.30 mg/ml concentration was not used for cell attachment or mechanical characterization.

### Fibre formation through wet spinning

Fibres were spun from prepared solutions using two methods: shear extrusion and microfluidic spinning. Shear extrusion was accomplished according to a standard method^55^ where silk dope is flown through a narrow needle into a coagulation bath. Microfluidic spinning is an adaptation of shear extrusion and was performed as previously documented^12^. Both methods have been reported to give similar mechanical properties (Fig. S4)^12^,^43^. In both cases, silk dope was loaded into a 250 μl gastight syringe (Hamilton Company, Reno, NV) and pumped through 23 G needles (Becton Dickinson, Franklin Lakes, NJ) either directly into a coagulation bath or first into the microfluidic device and then into a coagulation bath. The coagulation solution was 70% methanol (Fisher Scientific, Pittsburgh, PA) for microfluidic spinning in order to enable lateral extrusion by hastening fibre formation times. 90% isopropanol (Sigma-Aldrich, St. Louis, MO) was used during shear extrusion to allow for vertical flow, which prevents early coagulation from blocking steady flow^56^. Mature fibres remained in their respective coagulation bath for 1 minute after extrusion before being collected around a mandrel with a rotation speed equivalent to the exit speed of the solution to prevent post-spin stretching. Fibres used for mechanical testing were collected exclusively using the microfluidic spinning technique due to the facility of predicting and generating a wide range of diameters by modulating the flow rate of the silk dope relative to that of the focusing solution of acidified poly(ethylene oxide)^12^. Fibres used for all other experiments were collected using the shear extrusion method. Fibres used for cell culture were additionally washed multiple times in sterile PBS. Control fibres were produced by following this same process but with the SF lacking any added Fn.

### Fluorescence imaging

Fibres with three fluorescently labelled Fn doping concentrations (0.05, 0.15, 0.30 mg/ml) were placed on 12% polyacrylamide (PAA) gels, submerged in water, and imaged using an IX81 light microscope (Olympus, Center Valley, PA) in order to determine the uptake of the Fn into the SF fibre both in terms of quantity and distribution. Total fluorescent signal was recorded for multiple fibres, which was normalized by total fibre volume after removing background. Fibres greater than 20 μm in diameter were rejected from analysis in order to minimize noise introduced from out-of-focus fluorescent elements.

### Fibre physical properties characterization

To obtain tensile properties, alloy fibres with a Fn concentration of 0.15 mg/ml and control SF fibres were set onto sacrificial frames using two-sided tape in order to transfer from the spinning platform to the mechanical testing apparatus. Diameters were measured using the IX81 after which fibres were loaded onto a custom-built tissue stretcher capable of detecting forces reliably in the 0.1–100 mN range^57^. Once the frames were loaded onto the device, the edges were burned away and extension to failure tests were performed either in ambient air with 20% humidity or submerged in a room temperature water bath. For the ambient air environment, the strain rate was 0.01 s^−1^. Because changes in strain rate of soft, biological materials only lead to mechanical differences when the magnitude is modulated by orders of magnitude^58^, the hydrated environment employed a nominally higher strain rate of 0.05 s^−1^ in order to normalize the time to failure of the wet and dry fibres without risking modification to the stress-strain relationship. Stress-strain curves were generated and analysed to give values for the Young’s modulus, extension to failure, ultimate strength, and total material toughness for the four conditions tested. Young’s modulus was calculated by finding the slope of a linear regression to the initial elastic stretch region of the tests, extension to failure and ultimate strength were both taken directly from the curve, and toughness was calculated by finding the area under the stress-strain curve using trapezoidal summation.

### Cell culture and overnight adhesion assay

Cellular adhesion to the alloy and control fibres was tested by placing spun fibres onto a 12% PAA gel (Bio-rad Life Science, Hercules, CA) cast covalently onto a silanised 30 mm glass coverslip (Warner Instruments, Hamden, CT) inside a 1-cm tall custom-made culture chamber. PAA gels were used to support fibres for cell-based experiments due to their characteristic non-fouling and biologically inert surfaces^59^,^60^. NIH 3T3 fibroblasts, primary bovine aortic endothelial cells (BAEC), and primary bovine vascular smooth muscle cells (BVSMC) were cultured using Dulbecco’s modified eagle medium (DMEM) with 1.0 g/L glucose (Life Technologies) and enriched with 10% fetal bovine serum (FBS) (HyClone, Logan, UT). Once confluent, cells were passaged and spun down to remove all media. The cells were resuspended in serum-free DMEM and seeded onto PAA gels with either control or alloy fibres (0.05 mg/ml Fn concentration) on the surface at a density of 7,500 cells/cm^2^. The fibres with cells present were incubated for 24 hours. Images were taken of the fibres before and after a PBS wash, which removed debris and unattached material from view. Quantification of cellular adhesion was done by observing each attachment locus along the length of the fibres rather than by counting number of cells attached. For each attachment locus, the site was characterized as either a cell cluster, with more than one cell attached to the same location, or as an individual cell on the fibre.

### Cell migration on fibres and viability analysis

The 3T3s, BAECs, and BVSMCs were also seeded using the same procedure as explained in the previous section but instead resuspended in 10% FBS serum-enriched media and seeded at a density of 2,500 cell/cm^2^ to facilitate individual cell tracking. Alloy fibres were doped with 0.75 mg/ml Fn to more closely approximate pure Fn fibres, which exhibit high degrees of cell spreading and migration after attachment. Cells were allowed to attach to control and alloy fibres for 1 hour after seeding and then were placed inside a microscope-mounted cell culture chamber for visualization. Attached cells were found and imaged every 10 minutes for 12 hours for each cell type and both fibre conditions. Images were analysed to quantify the maximum cell spread length and 1-dimensional (1D) migration components of displacement, directional persistence, and magnitude of movement. Viable tracked cells were characterized as cells that did not die or detach terminally. Cell death was defined as cells that would ball up into rounded morphology while attached to the fibre and then visibly burst, as shown in Fig. S5. Terminal detachment was defined as a cell detaching while never reattaching to the fibre during the observation period.

### Statistical Analysis

The significance of observed differences in material properties between the alloy and control SF fibres was determined using a two-tailed Student’s t-test with unequal sample variance. Differences were considered significant in the 95% confidence interval (*P* < 0.05). For survival curve statistical significance, a log-rank test was applied to the data using the same confidence interval. Data with error bars are presented in figures as mean ± standard error of the mean. P-values are reported for all experiments with statistical significance comparisons.

## Additional Information

**How to cite this article:** Jacobsen, M. M. *et al*. Silk-fibronectin protein alloy fibres support cell adhesion and viability as a high strength, matrix fibre analogue. *Sci. Rep.* **7**, 45653; doi: 10.1038/srep45653 (2017).

**Publisher's note:** Springer Nature remains neutral with regard to jurisdictional claims in published maps and institutional affiliations.

## Supplementary Material

Supplementary Information
