# Supplementary material for: Silk-fibronectin protein alloy fibres support cell adhesion and viability as a high strength, matrix fibre analogue
Source: Sci Rep. 2017 Apr 5;7:45653. doi: 10.1038/srep45653 (PMC5381220; doi:10.1038/srep45653)
Supplement: Supplementary Information [file srep45653-s1.pdf]

Silk-fibronectin protein alloy fibres support cell adhesion and viability as a high strength, matrix fibre analogue

Matthew M. Jacobsen, David Li, Nae Gyune Rim, Daniel Backman, Michael L. Smith, Joyce Y. Wong

Supplementary Information

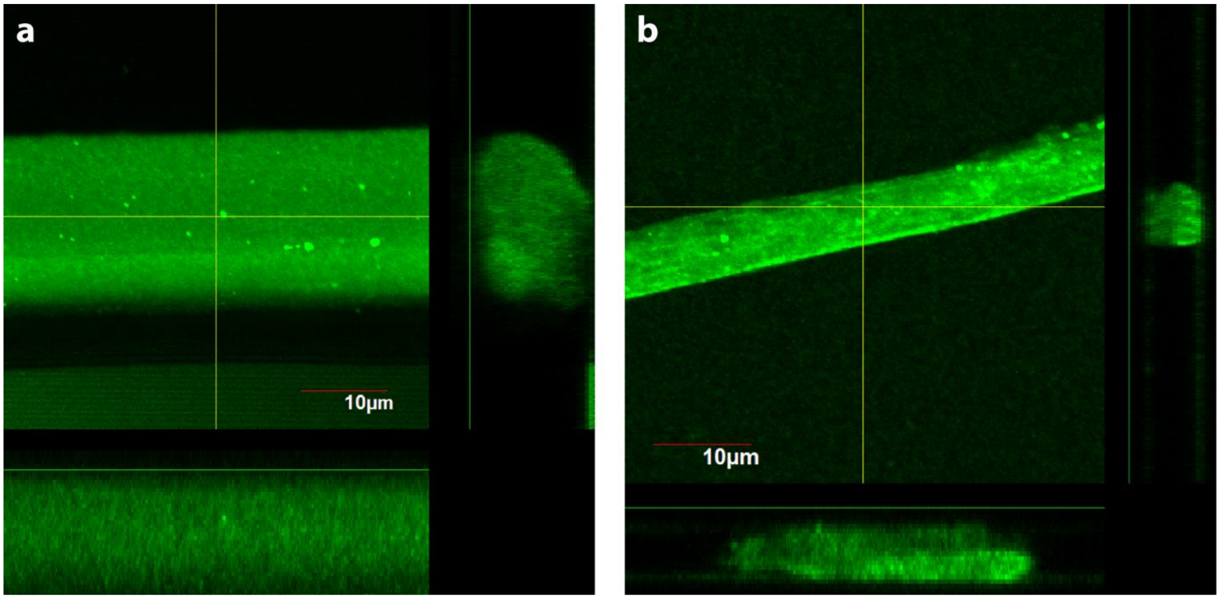

Figure S1: Confocal observation of SF-Fn alloy fluorescence. a, Confocal sum of fluorescence of the z-series probing a 20 μm diameter alloy SF-Fn fibre with 0.05 mg/ml Fn concentration. The cross-section of the fibre through the horizontal yellow line is shown below while the cross-section of the vertical yellow line is shown to the right. Fluorescence is seen throughout with relative uniformity, agreeing with the epifluorescence images of the lower Fn concentrations. **b,** Similar images shown for an 8 μm fibre with 0.30 mg/ml incorporated Fn. Laser power is reduced six times to compensate for the greater signal. Signal is similarly seen throughout the fibre and its cross-sections; however, the distribution is much more heterogeneous, also agreeing with the epifluorescence images showing that the increasing quantities of Fn reduce the uniformity of its distribution. The z-resolution of the confocal objective used is 946 nm with z-stack slices being 460 nm apart.

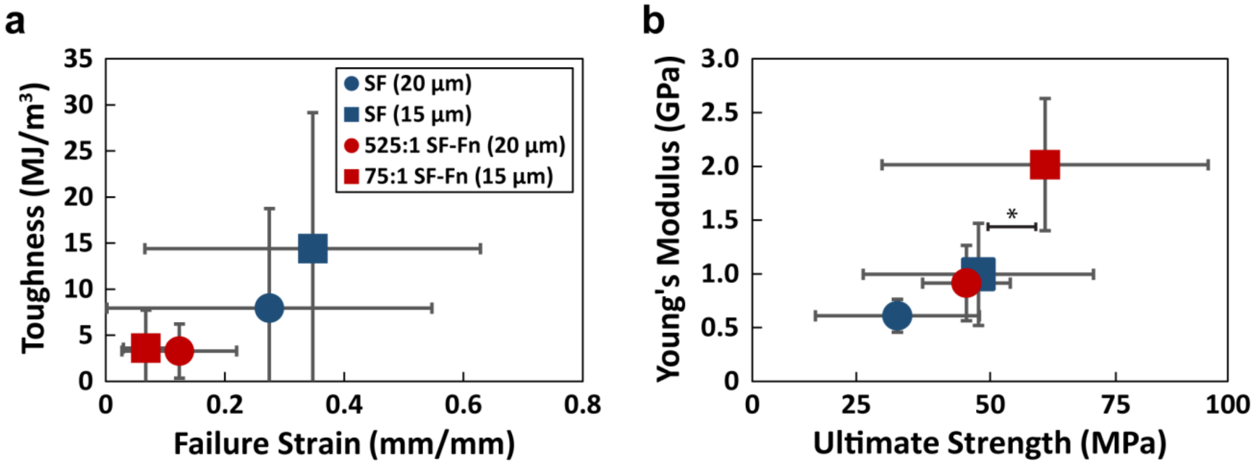

Figure S2: Mechanical trend of modifying the SF:Fn alloy ratio. Fibres were spun from the 75:1 SF:Fn ratio using the microfluidic method and mechanically tested to compare to control SF and the 525:1 ratio alloy fibres (0.15 mg/ml Fn incorporated). **a,** The observation that Fn incorporation reduces the extensibility of the SF-Fn fibres appears to be a trend with the 75:1 fibres having the lowest average failure strain. Notwithstanding, none of the changes observed among the failure strain and toughness values demonstrate statistical significance due to the large variability of the material properties, although the difference in extension to failure is close to qualifying between 75:1 SF-Fn fibres and equal diameter (15 µm) control SF fibres (P = 0.090). **b,** Similarly, the strength and stiffness of alloy fibres increases further with more Fn incorporated in the 75:1 ratio alloy fibre, which even have a statistically significant difference in Young's modulus compared to 15 µm diameter SF fibres (P = 0.020). Error bars represent one standard deviation with N=5 for all cases.

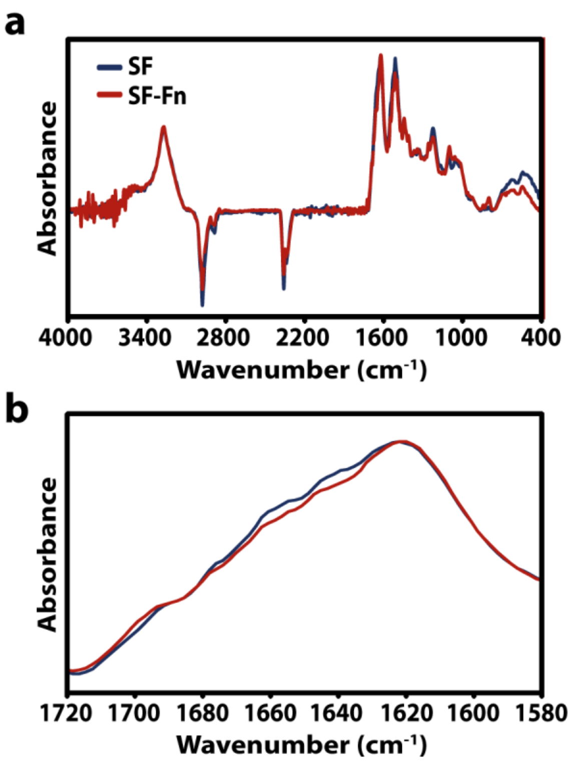

Figure S3: FT-IR spectra of the alloy and control fibres. a, Observation of the total unmodified spectrum (4000-400 cm^{-1}) shows little variation in composite structure. **b,** Focus on the amide I band demonstrates a slight difference in the approaching slopes to the local peak at 1620 cm^{-1} where both spectra exhibit their maximum value. This is indicative of a minor relative gain in β-sheet content compared to other secondary forms, which would infer β-strand exchange between the silk and fibronectin proteins.

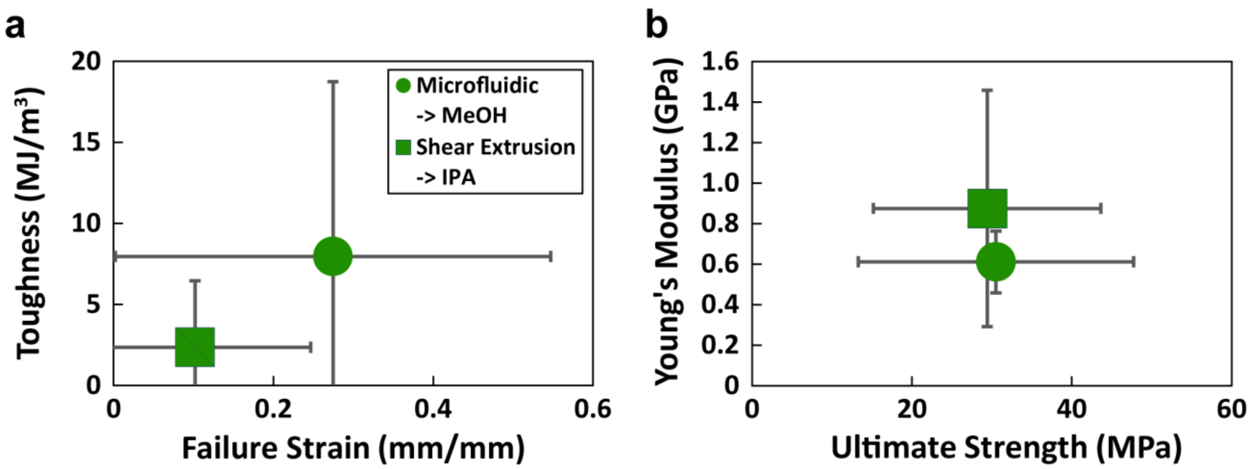

Figure S4: Mechanical comparison of shear to microfluidic spinning. Fibres were spun using both shear extrusion and microfluidics and those with diameters near 20 μm were tested to compare their mechanical properties. **a,** The properties that were the most different were the failure strain ($P = 0.26$) and toughness (P = 0.33), but neither of these are significant changes mostly due to the highly variable nature of the strain metric. **b,** The Young's modulus ($P = 0.38$) and, most especially, the ultimate strength ($P = 0.92$) show even greater similarity. These values provide further support for the prior evidence of other investigations that demonstrate these two methods as producing mechanically similar fibres. Error bars represent one standard deviation with N=5.

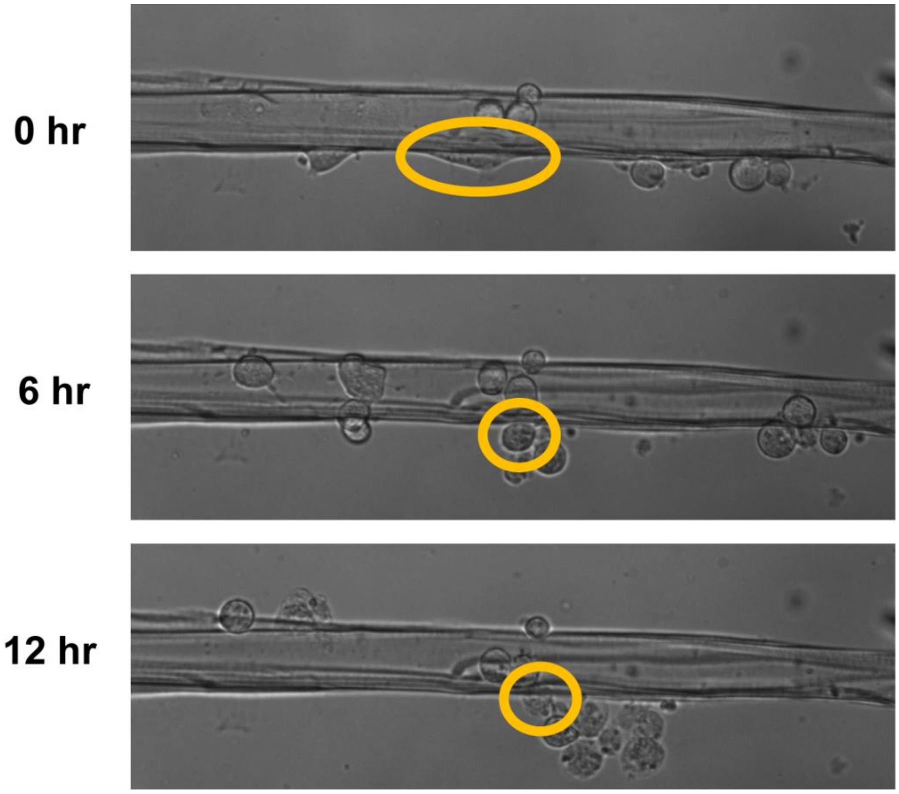

Figure S5: Criteria for cell death. Cell death was monitored optically and two criteria were required to qualify as a death. First, the cell must undergo a rounding of its morphology, as seen from hours 0 to 6. Then, the integrity of the cell membrane must be unequivocally compromised, often seen as a sudden burst leaving behind debris of the cell bound to the fibre, surrounding cells, or both.

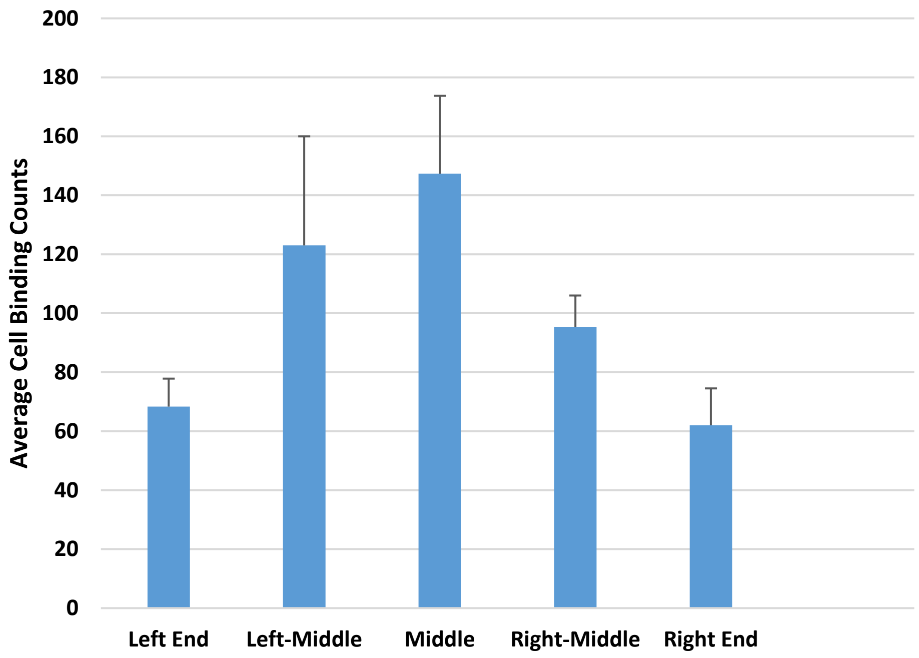

Figure S6: Histogram of cell binding based on position along the fibre on PAA gels. Cell binding to the fibre is reported as an average of all interactions along the length of the fibre. However, there is unequal binding to the fibres based on position. Here, the fibre is binned into 5 equal lengths. The average length of a whole fibre analysed was 17.5 ± 1.15 mm so each bin represents approximately 3.5 mm of the fibre length. This demonstrates an artefact of the attachment investigations caused by the non-homogeneous shape of the gel on which the fibres are deposited. The centre area of the circular gel is depressed slightly compared to the edges, leading to concentrations of cells accumulating towards the centre, which results in a greater number interacting with the fibres in that region.

Table S1: Full list of calculated values from the mechanical analysis

Dry RSF

Fiber	Diameter (µm)	Max Strain	Max Stress (MPa)	Modulus (GPa)	Toughness (MJ/m^3)
1	21.7	0.046	18.37	0.546	0.500
2	20.0	0.498	28.50	0.472	11.226
3	32.4	0.317	12.14	0.217	2.491
4	18.9	0.049	31.38	0.847	0.893
5	12.3	0.518	85.07	1.423	33.589
6	16.4	0.632	58.91	0.676	25.563
7	32.8	0.074	12.59	0.313	0.584
8	34.6	0.199	9.72	0.152	1.542
9	24.2	0.148	15.40	0.514	1.595
10	11.7	0.039	33.85	1.563	0.805
**Average**	**22.5**	**0.252**	**30.59**	**0.672**	**7.879**
**StanError**	**2.7**	**0.071**	**7.65**	**0.152**	**3.801**

Wet RSF

Fiber	Diameter (µm)	Max Strain	Max Stress (MPa)	Modulus (MPa)	Toughness (MJ/m^3)
1	31.8	2.306	5.69	9.482	6.697
2	29.8	2.296	5.18	12.314	7.117
3	11.4	1.967	25.44	40.565	25.925
4	32.8	1.698	5.74	9.960	5.487
5	13.2	2.080	21.08	18.152	20.225
6	23.7	3.329	5.31	5.786	11.178
7	17.4	1.802	9.14	7.123	8.466
8	26.9	1.612	5.06	6.024	4.264
9	22.5	2.525	6.48	12.014	9.291
10	19.5	3.766	12.14	14.723	27.056
**Average**	**22.9**	**2.338**	**10.12**	**13.614**	**12.571**
**Stan Error**	**2.4**	**0.223**	**2.32**	**3.238**	**2.707**

Dry RSF-Fn

Fiber	Diameter (µm)	Max Strain	Max Stress (MPa)	Modulus (MPa)	Toughness (MJ/m^3)
1	29.9	0.053	24.48	0.663	0.654
2	19.3	0.084	59.49	1.098	3.300
3	23.1	0.048	24.79	0.664	0.557
4	21.8	0.287	44.62	0.481	8.239
5	40.0	0.093	12.25	0.272	0.801
6	27.8	0.085	25.22	0.392	0.861
7	20.4	0.132	37.32	0.730	2.798
8	20.8	0.067	36.74	0.866	1.164
9	18.1	0.048	46.90	1.397	1.008
10	37.5	0.041	14.80	0.417	0.332
**Average**	**25.9**	**0.094**	**32.66**	**0.698**	**1.971**
**Stan Error**	**2.4**	**0.023**	**4.74**	**0.110**	**0.763**

Wet RSF-Fn

Fiber	Diameter (µm)	Max Strain	Max Stress (MPa)	Modulus (MPa)	Toughness (MJ/m^3)
1	44.2	1.543	2.89	9.050	2.995
2	17.4	1.650	20.41	38.155	21.138
3	37.5	3.674	4.34	9.662	8.873
4	47.9	2.373	2.70	7.349	3.946
5	37.0	2.109	2.98	4.706	3.600
6	20.9	2.258	13.20	25.689	17.118
7	18.1	2.306	11.72	33.719	17.976
8	13.9	0.877	18.18	31.668	8.151
9	11.5	1.653	35.38	79.786	42.044
**Average**	**27.6**	**2.049**	**12.42**	**26.643**	**13.982**
**Stan Error**	**4.7**	**0.258**	**3.66**	**7.892**	**4.173**
